# FPGA realization of four chaotic interference cases in a terrestrial trajectory model and application in image transmission

**DOI:** 10.1038/s41598-023-39823-x

**Published:** 2023-08-10

**Authors:** Miguel-Angel Estudillo-Valdez, Vincent-Ademola Adeyemi, Esteban Tlelo-Cuautle, Yuma Sandoval-Ibarra, Jose-Cruz Nuñez-Perez

**Affiliations:** 1 Instituto Politécnico Nacional, CITEDI, 22435 Tijuana, Mexico; 2 Instituto Nacional de Astrofísica, Óptica y Electrónica, INAOE, 72840 San Andrés Cholula, Mexico; 3 Universidad Politécnica de Lázaro Cárdenas, UPLC, 60950 Lázaro Cárdenas, Mexico

**Keywords:** Aerospace engineering, Electrical and electronic engineering

## Abstract

This article presents a technique to integrate two dynamical models, a four-wing spherical chaotic oscillator and the elliptical path described by the planet Earth during its translation movement around the sun. Four application cases are derived from the system by varying the dynamics of the chaotic oscillator and these can be applied in information encryption to transmit RGB and grayscale images modulated by CSK. Consequently, the three main contributions of this work are (1) the emulation of the trajectories of the planet Earth with chaotic interference, (2) the CSK modulation and image encryption in a master-slave synchronization topology, and (3) the CSK demodulation for decryption without loss of information with respect to the original information. The three contributions are based on VHDL code implementation. The results of the synchronization, encryption and decryption technique were verified by means of time series and the encrypted images showed a correlation less than − 0.000142 and − 0.0003439 for RGB and grayscale format, respectively, while the retrieved image shows a complete correlation with the image original. In this work, the co-simulations were performed between MATLAB/Simulink and Vivado, using the VHDL language on two FPGA boards from different manufacturers, namely, Xilinx Artix-7 AC701 and Intel Cyclone IV.

## Introduction

Modern telecommunications employ a combination of complex encryption techniques and represent the new emerging paradigms to improve the protection, efficiency, and flexibility of information. However, many challenges are still faced in the mathematical modeling and electronic design of security systems.

Due to the increase in the amount of data that is used through the Internet network and the current trend of a delicate handling of information, techniques have been developed for the encryption of data from chaotic systems, with the intention to prevent attacks or interception of information. The main advantage of this process is that it is relatively easy to obtain a carrier that is not capable of replicating unless the parameters, initial conditions and methodology used for its development are known exactly. So, chaos with these characteristics can be added to a data signal to keep it private or secure during transmission. Chaotic models generate waveforms sensitive to initial conditions, and initially close trajectories tend to diverge exponentially from each other. Due to this characteristic, they have various applications in science. Chaos theory is widely studied in multiple areas such as telecommunications, medicine, astronomy, biology, prediction of natural events, among others^1–3^. These systems are based on differential equations that require certain parameters and initial conditions, without which the generated signal cannot be replicated.

In recent years, different methods for data encryption using chaotic systems have been reported in the literature. The work in^4^ presents the implementation of chaotic models that are selected by users who wants to change their behavior or mix them with a map of chaotic points to improve data encryption. In^5^, fundamental research is carried out to be able to mix a chaotic signal with an elliptical trajectory, and since this is the typical shape of a planetary orbit, its methods are very useful. The investigation in^6^ presents a non-coherent chaotic modulation and demodulation technique, Differential Chaos Shift Keying (DCSK), and an Orthogonal Frequency-Division Multiplexing (OFDM) multi-user communication scheme, where the Bit Error Rate (BER) and the receiver is reported to outperform the conventional scheme for both Additive White Gaussian Noise (AWGN) and impulsive noise models, including Middleton class A noise. In^7^ a non-coherent Chaotic Shift Keying (CSK) modulation scheme is applied in a high-speed multicolor visible light communication transmission system. In^8^ a multi-tone technique for cryptography using chaos is developed that also allows Multiple Input Multiple Output (MIMO) communication. Moreover, there are electronic design techniques to implement various chaotic oscillators in various information encryption applications^9^.

On the other hand, there is the possibility of mixing the behavior of a chaotic generator with a natural system modeled from its equations. The addition of various dynamic models as an information encryption principle is a viable alternative, and that is why research such as^10^ proposes an image encryption algorithm based on the dynamics of a moving water flow that it mixes with chaotic waveforms capable of withstanding cryptanalysis such as brute force and differential attacks. Additionally, biological models such as the interaction of the electromagnetic flux between two brain neurons are modeled in^11^ with the aim of achieving image encryption. In other investigations^12,13^, the principles of deoxyribonucleic acid (DNA) have been used to generate pseudo-random sequences for the encryption of images in RGB and monochrome format, respectively, and which is also effective against noise and capable of resisting various typical attacks. Additionally, the use of Field-Programmable Gate Arrays (FPGAs) for digital implementations is very useful due to the flexibility that these devices allow, in addition to the greater data processing capacity^14,15^. The advantage of using the FPGAs is that the systems can be simulated prior to an analog or digital implementation, reducing manufacturing costs and time.

The results obtained in the previous investigations highlight the possibility of making mixtures between different dynamic models and thereby being able to grant a greater variety of parameters and sensitivity to the system. In this present investigation, we work with a dynamical model of the Earth’s orbit, adding chaotic interference signals from a four-winged spherical oscillator to an elliptical path^16^, based on the two-body problem in celestial mechanics that establishes the principles of interaction for any body suspended in space, where the body with less mass tends to orbit around the one with greater mass. The authors show that the generated waveforms are sinusoidal signals with chaotic interference due to the nature of the background planetary pattern, and are useful for signal encryption applications. Therefore, this article highlights the following contributions to the state of the art:

(i) FPGA-board realization of four cases of the Earth trajectories with chaotic interference. The architecture was designed using VHDL language with 40-bit word length on cards from two manufacturers, namely, Artix-7 AC701 from Xilinx and the Cyclone IV from Intel. Numerical simulations using Matlab are consistent with the VHDL implementation;
(ii) FPGA implementation of Hamiltonian synchronization with master-slave topology for CSK modulators and demodulators. The synchronization provides the needed backbone for the communication platform in the encryption and decryption of digital information with carriers based on Earth orbit with chaotic interference;
(iii) FPGA implementation of a secure communications system for image transmission using chaotic interference in the Earth orbit model. The system consists of the transmitter and receiver, to encrypt and recover an RGB image and another in grayscale format, respectively. Matlab numerical simulations and VHDL implementation of image transmission are in agreement.

This article is organized as follows, Section “Methods” presents the mathematical model of the four-wing oscillator, the model of the Earth’s orbit, the four interference cases, and the Hamiltonian synchronization method. Section “VHDL implementation and system co-simulation” describes the implementation in VHDL language and the co-simulation of the proposed system. Section “Application in image transmission” details the secure communications system for image transmission, with the application of the four cases of chaotic interference in the path of planet Earth to decipher RGB and grayscale images. Section “Results and discussion” contains the discussion of the results in terms of logical resources. Finally, Section “Conclusions” presents the conclusions.

## Methods

This section describes the relevant concepts and techniques in this investigation: four-wing oscillator, Earth orbital trajectory model, four cases of chaotic interference, and Hamiltonian synchronization.

### Four-winged spherical chaotic oscillator

**Figure 1:**
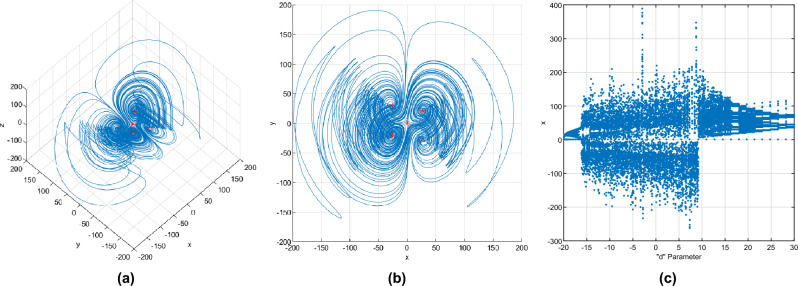
Four-wing spherical chaotic oscillator, (**a**) $x-y-z$ plane, (**b**) $x-y$ plane, (**c**) Bifurcation diagram for the *d* parameter.

The system of differential equations of Eq. (1) models a four-winged attractor with chaotic trajectories^16^,

1 $$\begin{aligned} \begin{matrix} {\dot{x}} &{}= ax + by + cyz \\ {\dot{y}} &{}= dy - xz \\ {\dot{z}} &{}= ez + fxy \end{matrix} \end{aligned}$$

where *x*, *y*, *z* represent the state variables, and *a*, *b*, *c*, *d*, *e*, *f* are the system parameters. According to^16^, to obtain chaotic behavior in trajectories, one option is to use the parameters $a=-14$, $b=5$, $c=1$, $d=16$, $e=- 43$, $f=1$, although these values can vary according to a bifurcation diagram. The initial conditions were arbitrarily selected as $x(0)=1$, $y(0)=1$, and $z(0)=1$. These initial conditions can be any value except for the equilibrium points that are generated. The chaotic system (1) has five equilibrium points $E_P$: $E_{P1} = 0, 0, 0$; $E_{P2} = 26.2298, 20.7772, 12.6740$; $E_{P3} = -26.2298, -20.7772, 12.6740$; $E_{P4} = 26.2298, -28.9740, -17.6740$; and $E_{P5} = -26.2298, 28.9740, -17.6740$. The chaotic trajectories generated by the system (1) and the equilibrium points of the system are shown in Fig. 1. Figure 1c shows the bifurcation diagram that shows the behavior of the system while the control parameter *d* is varied in a range of [− 20, 30].

It can be seen that as the control parameter *d* varies between − 16 and approximately 25, the possible paths that the waveforms can take vary in a greater range of amplitudes. This property is desirable to generate signals with chaotic characteristics. The fourth-order Runge Kutta numerical method was used for the simulations in Matlab version R2020b, with a sampling step of 0.001 and a simulation time of 0.1235 s.

### Earth’s elliptical orbital path model

**Figure 2:**
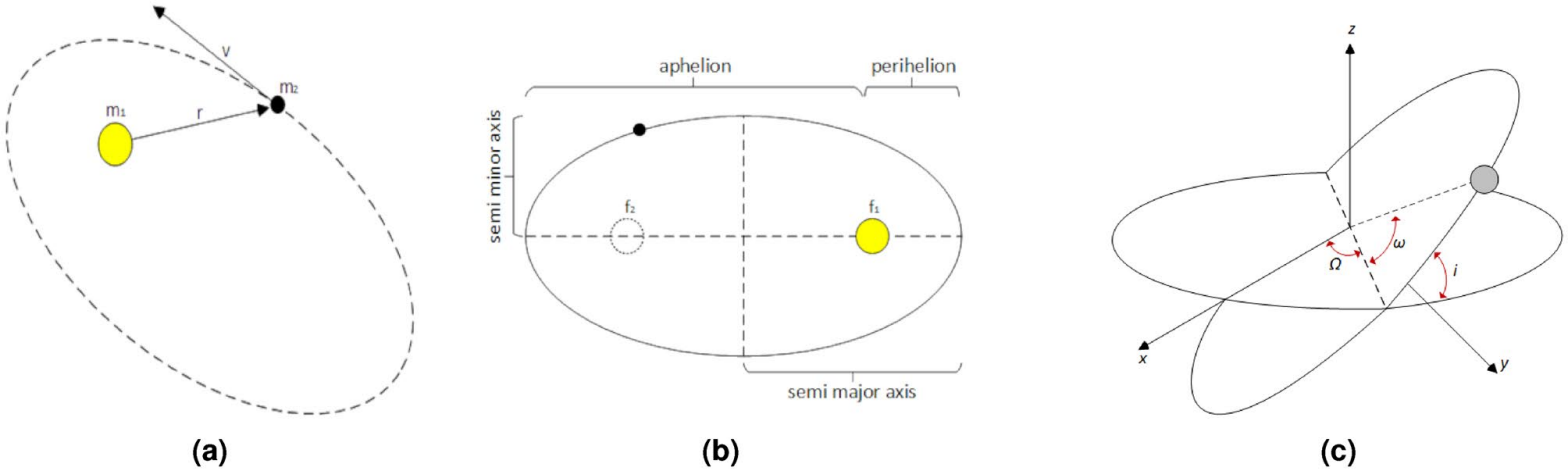
Two-body problem (**a**) trajectory of the secondary body in relation to the primary body (left), (**b**) representation of aphelion, perihelion, foci, semi-minor axis and semi-major axis (center), (**c**) Euler angles diagram (right).

This model is based on the two-body problem, which focuses on the behavior of the trajectories of two bodies that interact with each other through their gravitational attraction in space without external disturbances. Although every body in space is in motion, it is possible for practical purposes to establish that the body with the lower mass orbits around the one with the greater mass and this in turn is static. Numerically, it is possible to simulate the trajectories of planets or asteroids considering their gravitational force. Figure 2a shows the diagram of the trajectory of a secondary body orbiting the primary body, where the secondary body corresponds to the one with the lower mass and the primary body is the one with the greater mass. In Fig. 2b it is observed that the aphelion is the distance from the primary body to the farthest point of the elliptical path described by the secondary body, while the perihelion corresponds to the shortest distance of the ellipse. The eccentricity defines how circular an orbit is, and the greater the eccentricity the more elongated the orbit. So, an eccentricity of zero corresponds to a circular path. On the other hand, Fig. 2c shows the angles that locate the secondary body within the translational orbit.

**Figure 3:**
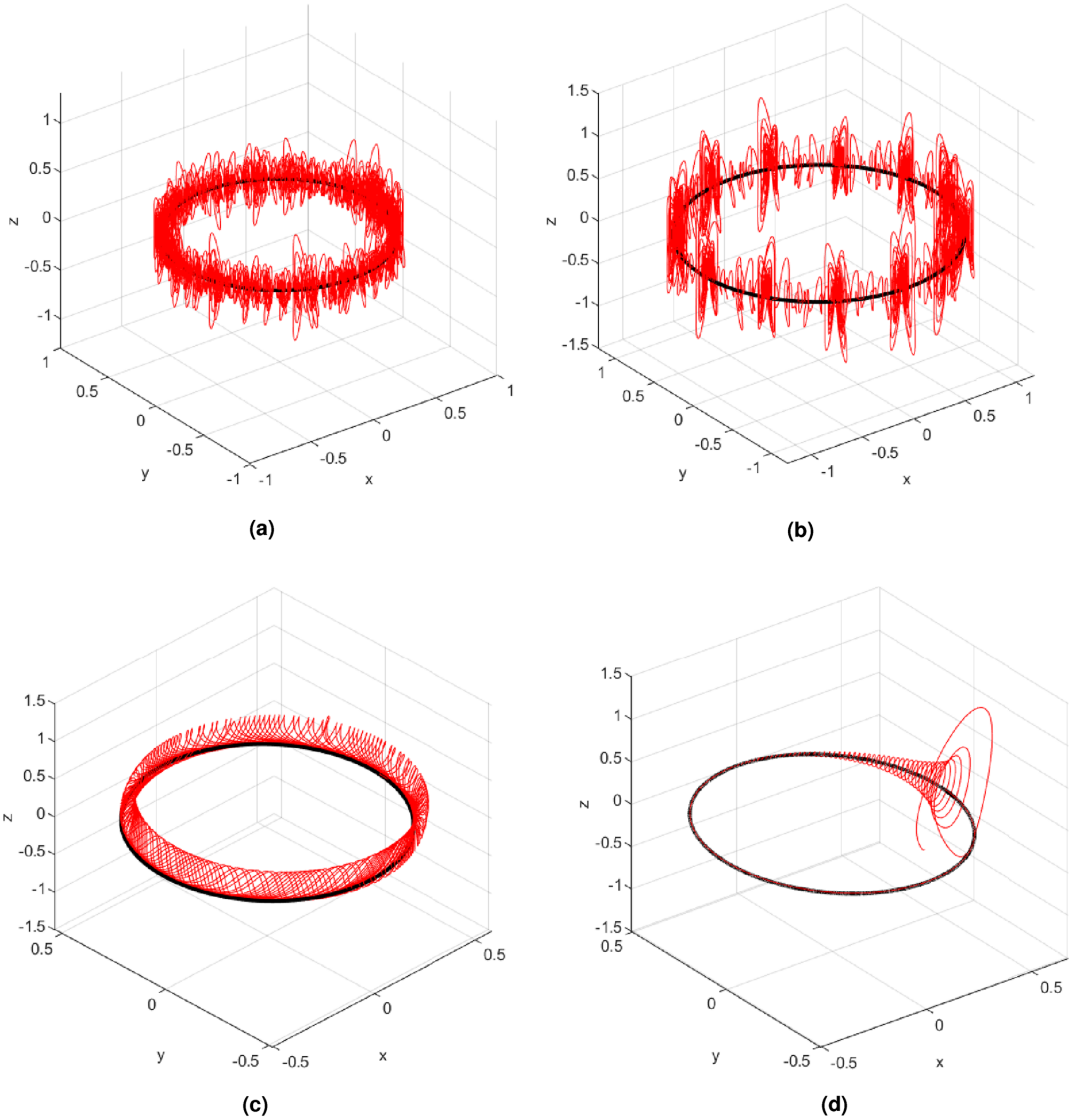
Elliptic path with chaotic interference for (**a**) Case 1, (**b**) Case 2, (**c**) Case 3 and (**d**) Case 4, in $x-y-z$ plane view.

The interaction between two bodies in space is carried out through an elliptical movement in which there are two foci within the ellipse. The body with the greater mass will be at one of the foci and the secondary body will move describing the ellipse as long as the gravitational and centrifugal forces of attraction of the body are balanced. The system of Eq. (2) determines the position described at each moment by a moving body within an elliptical path^5^.

2 $$\begin{aligned} \begin{bmatrix} x_r\\ y_r\\ z_r \end{bmatrix} = R_{xq} \begin{bmatrix} a(cos(E)-e)\\ \\ a\sqrt{1-e^2}sen(E)\\ \\ 0 \end{bmatrix} \end{aligned}$$

where $[x_r,y_r,z_r]$ are the spatial coordinates of the moving body, *a* represents the length of the semi-major axis of the orbit, *e* corresponds to the eccentricity of the elliptical orbit, *E* is the eccentric anomaly consisting of the angle measured from the center of the ellipse to the position of the secondary body, and $R_{xq}$ represents the rotation matrix^5^ that gives the inclination to the orbital plane, which is shown in Eq. (3),

3 $$\begin{aligned} \small R_{xq} =\begin{bmatrix} cos(\Omega )cos(\omega )-sin(\Omega )sin(i)sin(\omega ) &{} -cos(\Omega )sin(\omega )-sin(\Omega )cos(i)cos(\omega ) &{} sin(\Omega )sin(i)\\ cos(\Omega )cos(\omega )+cos(\Omega )cos(i)sin(\omega ) &{} -sin(\Omega )sin(\omega )+cos(\Omega )cos(i)cos(\omega ) &{} -cos(\Omega )sin(i)\\ sin(i)sin(\omega ) &{} sin(i)sin(\omega ) &{} cos(i)\\ \end{bmatrix} \end{aligned}$$

where $\Omega , \omega , i$ correspond to the Euler angles shown in Fig. 3c, which are used to locate a body while it describes an elliptical path. On the other hand, the velocity vector is represented by the system of Eq. (4), which allows determining the velocity of the body at each moment of its trajectory^5^.

4 $$\begin{aligned} \begin{bmatrix} {\dot{x}}_r\\ {\dot{y}}_r\\ {\dot{z}}_r \end{bmatrix}&= R_{xq} \begin{bmatrix} -\frac{nasen(E)}{1-ecos(E)}\\ \\ \frac{na\sqrt{1-e^2}cos(E)}{1-ecos(E)}\\ \\ 0 \end{bmatrix} \end{aligned}$$

where *n* is the orbital frequency and the rest of the variables correspond to Eq. (2).

The initial positions of the orbital model of the planet Earth $(x_r, y_r, z_r)$ are obtained using Eq. (2) and whose values of the main orbital and geometric parameters are mass${=5.97\times 10^ {24}}$ kg, aphelion ${= 816.62\times 10^{9}}$ km, perihelion ${= 102.94719}^\circ$, orbital inclination${=0.0005}^\circ$, ascending node longitude ${= - 11.26664}^\circ$.

### Four cases of chaotic interference in the terrestrial elliptical trajectory model

From the system of Eq. (1) it is possible to create a fusion between a chaotic attractor of (1) and the movement of a secondary body (4) by adjusting the equations of the chaotic oscillator of four wings to accommodate the position and speed of the secondary body using an elliptical orbit as its parameters. In this way, a chaotic trajectory is obtained that is modeled from a route defined by a natural system^5^. With this, the system of Eq. (5) is obtained that allows mixing both trajectories and generating waveforms with chaotic interference,

5 $$\begin{aligned} \begin{matrix} {\dot{x}} = a(x-x_r) + b(x-x_r) + c(y-y_r)(z-z_r) \\ {\dot{y}} = d(y-y_r)-(x-x_r)(z-z_r)\\ {\dot{z}} = e(z-z_r) + f(x-x_r)(y-y_r) \end{matrix} \end{aligned}$$

Setting the input angles of the rotation matrix as $\Omega =-11.26064^\circ$, $\omega =102.94719^\circ$, $i=0.00005^\circ$, and using Eq. (3), the values of $R_{xq}$ shown in Eq. (6),

6 $$\begin{aligned} R_{xq} =\begin{bmatrix} -0.2197 &{} -0.9996 &{} -1.7041 \times 10^{-7}\\ 0.7361 &{} -0.0294 &{} -8.5586 \times 10^{-7}\\ 8.5048 \times 10^{-7} &{} 8.5048 \times 10^{-7} &{} 1.0\\ \end{bmatrix} \end{aligned}$$

It is necessary to have the values of all the parameters to determine the characteristics of the elliptical orbit through which the chaotic behavior will develop. Manipulating the eccentric anomaly *E* of the ellipse as a set of points going from 0 to 2$\pi$ rad (0° to 360°) around the circumference of the ellipse in various units *L*, representing the total extent of the ellipse, and by the variation of the parameter of the spherical oscillator, four different cases are analyzed. Figure 3 illustrates the 3-D simulation plots of the four cases of chaotic interference in a four-winged oscillator, where the size of the Earth’s orbit was normalized for the four cases with a reduction factor of 5 × $10^{-12}$.

**Case 1:** *Chaotic trajectory along the orbit* The secondary body is the planet Earth that moves around the Sun describing an ellipse while chaos continuously adheres to the orbit. A continuous increment is established for the eccentric anomaly *E* from $(2\pi /1000)$rad until reaching $2\pi$rad (0° to 360°). The orbital frequency is obtained by Eq. (7),
  7 $$\begin{aligned} n =\frac{E-e {sin(E)}}{L} \end{aligned}$$
  where *n* represents the average movement of the secondary body, and the denominator *L* corresponds to the difference between the initial time $t_0$ and the final time $t_f$, that is, $L=t_f-t_0$. The values used in this case for the input parameters are $E = 2\pi$ rad and *L* = 200. By simulating the system, the attractor shown in three dimensions in Fig. 3a is obtained, where it is highlighted that the center of the secondary body moves along the elliptical orbit marked in black at the same time that the winding around it is highlighted in red. In the attractor of Case 1 it is not observed that there is the same shape of four wings that the main attractor presents. This is because the movement of the center of the secondary body is continuous through the ellipse at the same time that the second body rotates randomly, meaning that there is not enough time to create the attractor shape. To achieve a four-winged attractor, the center of the secondary body must stop at one point long enough.
**Case 2:** *Chaotic interval path* The secondary body temporarily stops at a point in the orbit while the chaos develops its waveforms. Subsequently, the secondary body continues its journey for a brief moment of time before stopping again while the trajectory continuously forms the four wings. Keeping the same parameters as in Case 1, Case 2 has the particularity of presenting a discrete angle for *E* that goes from 0 to 2$\pi$ rad (0° to 360°), where *E* jumps a certain number of radians between each location at the center of the secondary body during one full turn before changing to the next position. In Fig. 3b, it can be seen that the four-winged spherical chaotic attractor has several attached subattractors, where the end of one is the beginning of another as a result of the combination of a conservative celestial system (represented by the ellipse ) with a chaotic dissipative system, that is, the four-winged attractors. As seen at each lag point, the system spawns a four-wing chaotic subattractor and then jumps through the orbit an amount $\Delta$ $E$ = $\pi$ $/5 = 36^\circ$ to spawn another subattractor. Remembering that E = 2$\pi$, it is determined that the system generates a total of ten subattractors. It can also be seen that although the center of each subattractor is in the orbit, the transition between one and the other is not the same along the ellipse. This is due to the fact that the initial state of the subattractor does not find the same position.
**Case 3:** *Periodic orbits* The waveforms in this case are generated continuously along the elliptical path. The system’s orbit rotates periodically around its axis as it moves around the center of mass. The parameter $c=1$ is modified to $c=5$, changing the dynamics of the chaotic oscillator and its behavior from chaotic to periodic. As a result of this coiling, the trajectory of the secondary body generates a spring-like shape that wraps around the elliptical orbit as. This case is shown in Fig. 3c.
**Case 4:** *Converging elliptic trajectory* The parameter $c = 12$ is used so that the orbit of the system converges to an equilibrium point while making revolutions around the center of mass. When the velocity and position equations are established, maintaining the parameter $c = 12$, the system rotates around the central body and when it reaches its equilibrium point located in the sink, it converges to equilibrium producing an elliptical orbit from the sink. Figure 3d shows that the system makes revolutions around the equilibrium point and generates the ellipse. The system’s orbit starts at an initial point and converges to the equilibrium orbit, coupling the ellipse generated by the celestial system with the sink.

### Hamiltonian synchronization

This synchronization is based on a master-slave topology composed of two chaotic oscillators with identical parameters, but with different initial conditions^17^. Consider the nonlinear system in the generalized Hamiltonian form in Eq. (8).

8 $$\begin{aligned} {\dot{x}} = J(x) \dfrac{\partial {\varvec{H}}}{\partial x} + S(x) \dfrac{\partial {\varvec{H}}}{\partial x} \end{aligned}$$

where $\varvec{H(x)}$ describes a globally positive definite energy function of the form ${\varvec{H}}(x)=1/2 x^T Mx$ and the gradient vector of ${\varvec{H}}$ is denoted $\partial {\varvec{H}} / \partial x$. Also, the square matrices *J*(*x*) and *S*(*x*) satisfy $J(x)+J^T (x)=0$ and $S(x)=S^T (x)$. Considering a special class of generalized Hamiltonian systems with destabilizing vector fields, Eq. (9) is obtained,

9 $$\begin{aligned} \begin{matrix} {\dot{x}}_m = J(y) \dfrac{\partial {\varvec{H}}}{\partial x_m} + (I+S) \dfrac{\partial {\varvec{H}}}{\partial x_m}+ F(y), \hspace{0.2cm} x_m \in \mathbb {R},\hspace{0.2cm} y=C\dfrac{\partial H}{\partial x_m}, \hspace{0.2cm} y \in \mathbb {R}^n \end{matrix} \end{aligned}$$

where *C* corresponds to a constant symmetric matrix and *I* corresponds to a constant diagonal symmetric matrix. By selecting the variables $\xi$ and $\eta$ as state vector and vector estimated at the output, an observer system is generated from Eq. (9) represented by Eq. (10) with a vector *K* known as the observer gain vector,

10 $$\begin{aligned} \begin{matrix} {\dot{\xi }} = J(y) \dfrac{\partial {\varvec{H}}}{\partial \xi } + (I+S) \dfrac{\partial {\varvec{H}}}{\partial \xi } + F(y) + K(y-\eta ),\\ \end{matrix} \end{aligned}$$

where $\eta$ is represented by Eq. (11),

11 $$\begin{aligned} \eta = C\dfrac{\partial {H}}{\partial {\xi }} \end{aligned}$$

From (9) and (10) the synchronization process is developed in a structured manner.

First, the quadratic energy function shown in Eq. (12) is selected,

12 $$\begin{aligned} {\varvec{H}}(x) = \dfrac{1}{2}[ax^2 + by^2 + z^2] \end{aligned}$$

in turn the gradient vector of Eq. (12) is shown in Eq. (13),

13 $$\begin{aligned} \dfrac{\partial {\varvec{H}}}{\partial x} = \begin{bmatrix} ax \\ by \\ z \\ \end{bmatrix} = \begin{bmatrix} a &{} 0 &{} 0 \\ 0 &{} b &{} 0 \\ 0 &{} 0 &{} 1 \\ \end{bmatrix} \begin{bmatrix} x \\ y \\ z \\ \end{bmatrix}= {\varvec{P}}. \end{aligned}$$

Then the matrices *J* and *S* are obtained by means of the matrix representation of the chaotic oscillator modeled by the system of Eq. (1) resulting in Eq. (14),

14 $$\begin{aligned} A= \begin{bmatrix} a &{} b &{} cy \\ 0 &{} d &{} -x \\ fy &{} 0 &{} e \\ \end{bmatrix} \begin{bmatrix} x \\ y \\ z \\ \end{bmatrix}. \end{aligned}$$

Equation (14) is rewritten in terms of *J* and *S*, generating Eq. (15),

15 $$\begin{aligned} A = {\varvec{R}}\dfrac{\partial {\varvec{H}}}{\partial x} = [J(y) + S(y)]\dfrac{\partial {\varvec{H}}}{\partial x}, \end{aligned}$$

where *R* is an auxiliary matrix and when cleared, it takes the form of Eq. (16),

16 $$\begin{aligned} {\varvec{R}} = \varvec{AP^{-1}} = \begin{bmatrix} 1 &{} 1 &{} cy \\ 0 &{} \dfrac{d}{b} &{} -x \\ \dfrac{fy}{a} &{} 0 &{} e \\ \end{bmatrix}. \end{aligned}$$

From Eq. (16) we have the relations (17) and (18),

17 $$\begin{aligned} S(x) = \dfrac{1}{2}(R+R^T) = \begin{bmatrix} 1 &{} 1/2 &{} \dfrac{cy}{2}+\dfrac{fy}{2a} \\ \\ 1/2 &{} d/b &{} -x/2\\ \\ \dfrac{cy}{2}+\dfrac{fy}{2a} &{} -x/2 &{} e\\ \end{bmatrix}, \end{aligned}$$

18 $$\begin{aligned} J(x) =\dfrac{1}{2}(R-R^T) = \begin{bmatrix} 0 &{} 1/2 &{} \dfrac{cy}{2}-\dfrac{fy}{2a} \\ \\ -1/2 &{} 0 &{} -x/2\\ \\ \dfrac{fy}{2a}-\dfrac{cy}{2} &{} x/2 &{} 0\\ \end{bmatrix}. \end{aligned}$$

For this case, the matrix $C = [1 \ \ 0 \ \ 0]$ is considered.

Subsequently, the synchronization of the master-observer systems is carried out, the master system is obtained from developing Eq. (9) as shown in Eq. (19),

19 $$\begin{aligned} \small \begin{bmatrix} {\dot{x}}_m \\ {\dot{y}}_m \\ {\dot{z}}_m \\ \end{bmatrix} = \begin{bmatrix} 0 &{} \dfrac{1}{2} &{} \dfrac{cy}{2}-\dfrac{fy}{2a} \\ \\ -\dfrac{1}{2} &{} 0 &{} -\dfrac{x}{2}\\ \\ \dfrac{fy}{2a}-\dfrac{cy}{2} &{} \dfrac{x}{2} &{} 0\\ \end{bmatrix} \begin{bmatrix} ax \\ by \\ z \\ \end{bmatrix} + \begin{bmatrix} 1 &{} \dfrac{1}{2} &{} \dfrac{cy}{2}+\dfrac{fy}{2a} \\ \\ \dfrac{1}{2} &{} \dfrac{d}{b} &{} -\dfrac{x}{2}\\ \\ \dfrac{cy}{2}+\dfrac{fy}{2a} &{} -\dfrac{x}{2} &{} e\\ \end{bmatrix} \begin{bmatrix} ax \\ by \\ z \\ \end{bmatrix}. \end{aligned}$$

In the same way, the observing system is represented by Eq. (20),

20 $$\begin{aligned} \tiny \begin{bmatrix} {\dot{\xi }}_{1} \\ \\ {\dot{\xi }}_{2} \\ \\ {\dot{\xi }}_{3} \\ \\ \end{bmatrix} = \begin{bmatrix} 0 &{} \dfrac{1}{2} &{} \dfrac{cy}{2}-\dfrac{fy}{2a} \\ \\ -\dfrac{1}{2} &{} 0 &{} -\dfrac{x}{2}\\ \\ \dfrac{fy}{2a}-\dfrac{cy}{2} &{} \dfrac{x}{2} &{} 0\\ \end{bmatrix} \begin{bmatrix} ax \\ by \\ z \\ \end{bmatrix} + \begin{bmatrix} 1 &{} \dfrac{1}{2} &{} \dfrac{cy}{2}+\dfrac{fy}{2a} \\ \\ \dfrac{1}{2} &{} \dfrac{d}{b} &{} -\dfrac{x}{2}\\ \\ \dfrac{cy}{2}+\dfrac{fy}{2a} &{} -\dfrac{x}{2} &{} e\\ \end{bmatrix} \begin{bmatrix} ax \\ by \\ z \\ \end{bmatrix} + \begin{bmatrix} K_1 \\ K_2 \\ K_3 \\ \end{bmatrix} (y - \eta ). \end{aligned}$$

By multiplying the matrices of Eqs. (20) and (21) is obtained,

21 $$\begin{aligned} \begin{bmatrix} {\dot{\xi }}_{1} \\ \\ {\dot{\xi }}_{2} \\ \\ {\dot{\xi }}_{3} \\ \\ \end{bmatrix} = \begin{bmatrix} \dfrac{by}{2}+\dfrac{cyz}{2}-\dfrac{fyz}{2a} \\ \\ -\dfrac{ax}{2}-\dfrac{xz}{2}\\ \\ \dfrac{fyax}{2a}-\dfrac{cyax}{2}+\dfrac{byx}{2}\\ \end{bmatrix} + \begin{bmatrix} ax+ \dfrac{by}{2}+\dfrac{cyz}{2}+\dfrac{fyz}{2a} \\ \\ \dfrac{ax}{2}+\dfrac{byd}{b}-\dfrac{xz}{2}\\ \\ \dfrac{cyax}{2}+\dfrac{fyax}{2a}-\dfrac{byx}{2}+ ez\\ \end{bmatrix} \begin{bmatrix} k_1(x_m-\xi _1) \\ k_2(y_m-\xi _2) \\ k_3(z_m-\xi _3) \\ \end{bmatrix}. \end{aligned}$$

Adding the matrices of Eq. (21) gives the compact observer system as shown in Eq. (22),

22 $$\begin{aligned} \begin{matrix} {\dot{x}} = ax + by + cyz + K_1(x_m - \xi _1)\\ {\dot{y}} = dy - xz + K_2(y_m - \xi _2)\\ {\dot{z}} = ez + fxy + K_3(z_m - \xi _3) \end{matrix} \end{aligned}$$

On the other hand, the synchronization will be considered complete when the phase difference between the channels of the master and slave system is zero, and when Theorems 1 and 2 are satisfied, according to^17^,

23 $$\begin{aligned} \lim _{t \rightarrow \infty }(||x_m-\xi _1||)=0 \end{aligned}$$

#### Theorem 1

*The state* $x_m$ *of the nonlinear system in Eq.* (12) *can be globally, exponentially, and asymptotically estimated by the state of the nonlinear observer* $\xi$ *of Eq.* (13) *if the pair of matrices* (*C*, *S*) *are observable*.

#### Theorem 2

*The state of* $x_m$ *in Eq.* (12) *can be globally, exponentially and asymptotically estimated by the state* $x_e$ *of the nonlinear observer in Eq.* (13), *if and only if there exists a constant matrix K such that the symmetric matrix*

24 $$\begin{aligned} \small [W-KC]+[W-KT]^T=[S-KC]+[S-KC]^T=2[S-1/2(KC+C^TK^T)] \end{aligned}$$

*be definite negative.*

**Figure 4:**
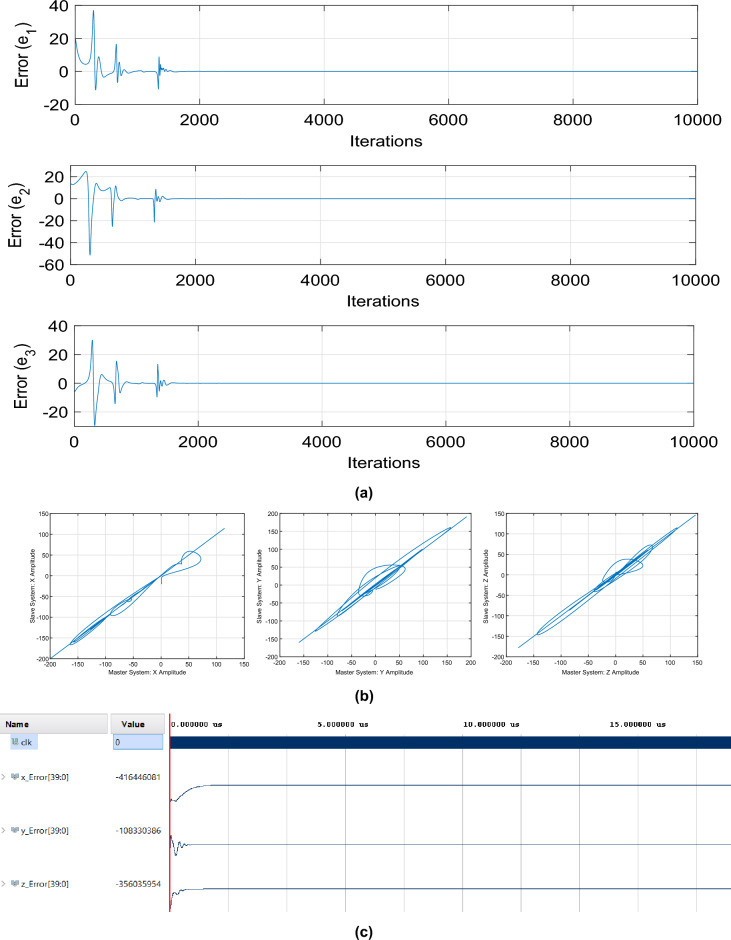
Synchronization between master-slave systems (**a**) synchronization errors in MATLAB, (**b**) phase errors in MATLAB, and (**c**) synchronization errors in Vivado.

In this work, the chosen gains of the observer are $k_{1}$ = 20, $k_{2}$ = 15 and $k_{3}$ = 10. The timing errors are calculated and plotted as $e_{ 1} = x_{1} - \xi _{1}$, $e_{2} = x_{2} - \xi _{2}$, and $e_{3} = x_{3} - \xi _{34}$ , where the master is $x_{i}$ and the slave is $\xi _{i}$. The phase error between $x_i$ and $\xi _{i}$ is also plotted. The total number of samples is $1 \times 10^{4}$. The Synchronization results are shown in Fig. 4. There are two significant aspects to consider: (1) the configuration, both in the transmitter and receiver, of the four-winged chaotic oscillator parameters. This process takes 110 microseconds, once the oscillator is set up; (2) the synchronization time, which takes an additional 1.2 microseconds using the four-winged chaotic oscillator mentioned in Eq. (1) and solved using the fourth-order Runge Kutta numerical method; and a clock period in the FPGA device of 10 nanoseconds. Therefore, for synchronization, at least 120 chaotic oscillator iterations are required, as a result, the total time from the beginning of the communication process until the first bit is transmitted is approximately 111.2 microseconds. Figure 4a shows the synchronization errors co-simulation plot in MATLAB /Simulink, where $e_{1}$, $e_{2}$ and $e_{3}$ are the synchronization errors, while the phase errors between master and slave system are shown in Fig. 4b. On the other hand, the stabilization of the error in the implementation of the synchronization in VHDL code using the Vivado software is shown in Fig. 4c. The error stabilization curves between Vivado and Matlab differ from each other, this is due to the arithmetic used for each case, where MATLAB applies a floating point arithmetic and in Vivado a fixed point arithmetic has been used due to the limitation of logical resources.

Consistent with the above paragraph, it can be seen that the first 111.2 µs of simulation are required for the system configuration and synchronization. After that time, each signal will begin the encryption and modulation process.

## VHDL implementation and system co-simulation

This section firstly describes the realization of the integrated system by a spherical chaotic oscillator in four cases of interference in the Earth’s orbit in a co-simulation between Matlab/Simulink and Xilinx’s System Generator for DSP in VHDL code blocks. In the FPGA implementation, two development boards were chosen, the Artix-7 AC701 from Xilinx and the Cyclone IV from Intel, both using the same code written in VHDL. For the calculations, 1 bit was used for the sign, 19 bits for the integer part, and 20 bits for the fractional part, since the use of fewer bits results in the loss of significant information after the calculations. In the realization, Cases 1 and 2 of chaotic interference and Cases 3 and 4 of periodic interference in the trajectory of the planet Earth are used to modulate and encrypte images, in RGB and grayscale formats.

**Figure 5:**
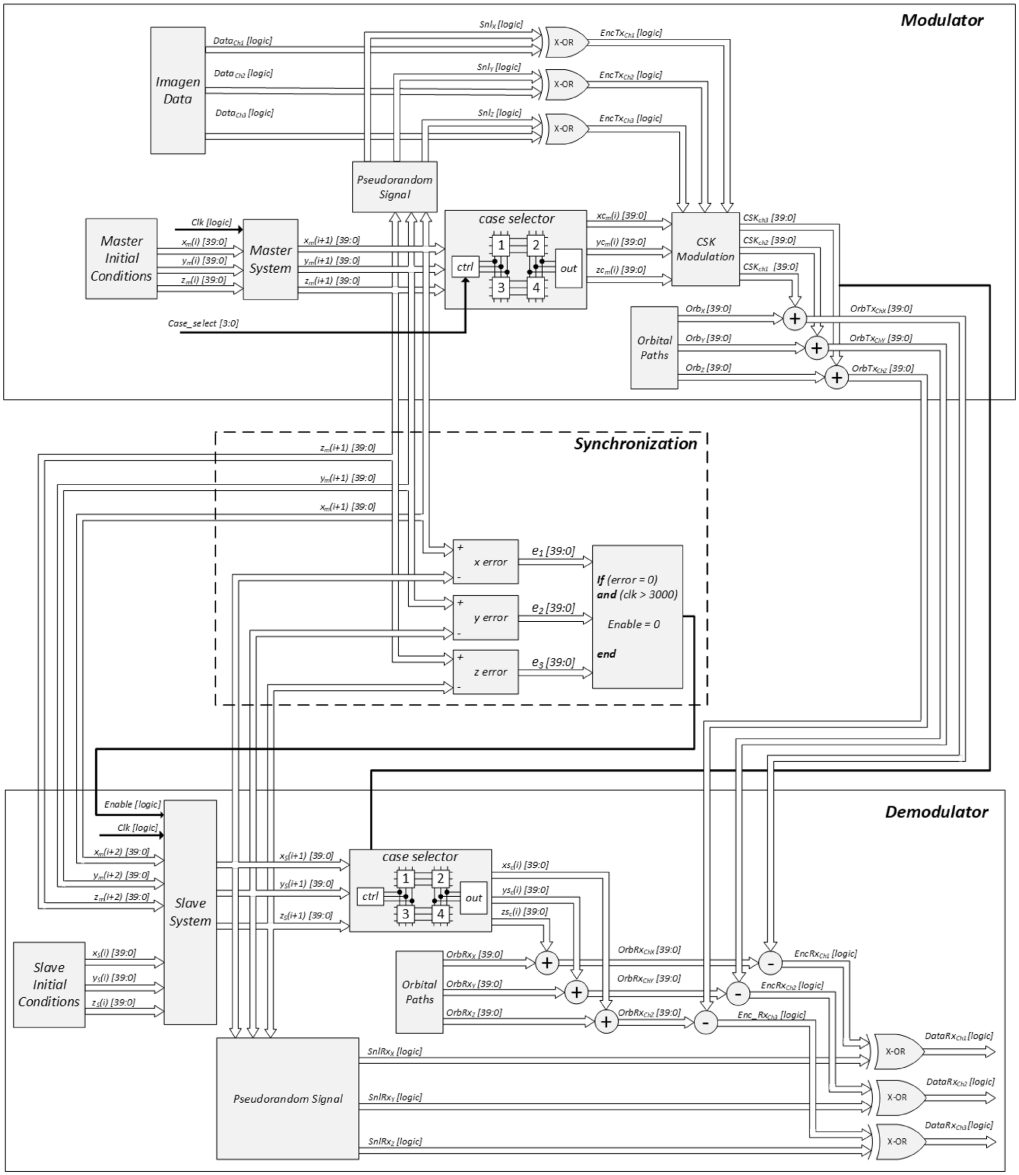
Block diagram of the encryption and modulation, demodulation and decryption process.

Figure 5 illustrates the flow diagram of the complete proposed system, where there is the transmitter, synchronization and receiver. The master system is located in the transmitter and the slave system is located in the receiver. The upper part shows the signal flow in the encryption and modulation process. At the center is the Hamiltonian synchronization with a master-slave topology, while in the lower part the flow of signals the demodulation and decryption process that allows the recovery of information is shown.

### Transmitter

In this work, any of the three variables *x*, *y*, *z* can be chosen as the transmission channel, obtaining $Channel_1, Channel_2, Channel_3$, respectively. That is, the channel signal $Data_{Ch1}$ corresponds to channel 1 using the variable *x*; the $Data_{Ch2}$ signal represents channel 2 using the *y* variable; and the signal of $Data_{Ch3}$ is channel 3 using the variable *z*, for information transmission. Subsequently, the initial conditions $x_m$, $y_m$, and $z_m$ of the master system are established, which allow generating its waveforms and follows three routes: (1) to a block that samples the chaotic waveforms to obtain three pseudo-random digital strings $Snl_x$, $Snl_y$, and $Snl_z$; (2) to a module that implements the chaotic interference case that will be used to modify the waveforms, generating the signals $xc_m$, $yc_m$, and $zc_m$ which become the carriers in the CSK modulation block; and (3) transmission to the receiver to use them in the synchronization of the master-slave system. The encryption of the input signal $Data_{Ch1}$ is carried out through a logical X-OR operation between the signals $Snl_x$ and their respective information channel $Data_{Ch1}$. The same happens for the information channels $Data_{Ch2}$ and $Data_{Ch3}$, obtaining the encrypted signals $EncTx_{Ch1}$, $EncTx_{Ch2}$, and $EncTx_{Ch3}$.

In this investigation, a CSK modulation was used due to the simplicity of the method with respect to its counterpart DCSK. The latter does not require synchronization to retrieve the information. However, the CSK technique has great robustness due to the low auto-correlation of chaotic carriers. In CSK modulation, the carrier is the chaotic signal, while the modulating signal is the message or information. The CSK modulated signal consists of a 180 degree inversion in the carrier signal when the modulating signal, converted to binary, corresponds to a low bit, and will not have inversion in degrees while the modulator remains in the high state. Therefore the output signals of the X-OR gate are used as the modulating signals in the CSK block, and the carriers will be the chaotic signals $$xc_m$$, $$yc_m$$, and $$zc_m$$, and the CSK modulated signals are generated $$(CSK_{Ch1}$$, $$CSK_{Ch2}$$, $$CSK_{Ch3})$$. Finally, the CSK modulated signals are added with the data of the Earth’s orbit $$( Orb_x$$, $$Orb_y$$, $$Orb_z)$$, generating the transmitter output signals $$(OrbTx_{ChX}$$, $$OrbTx_{ChY}$$, $$OrbTx_{ChZ})$$, which is sent to the receiver.

### Synchronization

The *Master* and *Slave* blocks represent the master and slave systems, respectively, while the *Sync* block handles the synchronization process. For the master system the outputs are $$x_m$$, $$y_m$$ and $$z_m$$, for the slave system the outputs are $$x_s$$, $$y_s$$ and $$z_s$$, while the synchronization errors are $$error_1$$ , $$error_2$$ and $$error_3$$. These blocks manage to correct the phase shift and find the synchrony between the signals of the master $$(x_m$$, $$y_m$$, $$z_m)$$ and the slave $$(x_s$$, $$y_s$$, $$z_s)$$. To do this, the value of the data signal of the slave $$[x_s(i+1)$$, $$y_s(i+1)$$, $$z_s(i+1)]$$ is subtracted from that of the master $$[x_m(i+1)$$, $$y_m(i+1)$$, $$z_m(i+1)]$$, and when the synchronization error value $$(error_1$$ , $$error_2$$, $$error_3)$$ is zero, it is considered that the synchronization has been successful and the original image information is transmitted. The master data $$[x_m(i+1)$$, $$y_m(i+1)$$, $$z_m(i+1)]$$ is sampled as pseudo-random signals $$(Snl_x$$, $$Snl_y$$, $$Snl_z)$$, which will be identical to those of the master in the transmitter and used to decrypt the information.

### Receiver

First, the case of interference selected in the transmitter is identified by means of a configuration signal coming from modulation channel 1, $$CSK_{Ch1}$$. This configuration process outputs the signals $$xc_m$$, $$yc_m$$, and $$zc_m$$ that are added to the values of the Earth’s orbits $$(OrbRx_x$$, $$OrbRx_y$$, $$OrbRx_z)$$. As a result of the sum the signals that represent the case of chaotic interference in the receiver are obtained $$(OrbRx_{Ch1}$$, $$OrbRx_{Ch2}$$, $$OrbRx_{Ch3})$$.

To obtain the transmitter information, the CSK modulated signals $$(OrbTx_{Ch1}$$, $$OrbTx_{Ch2}$$, $$OrbTx_{Ch3})$$ are subtracted from the chaotic interference waveforms generated in the receiver $$(OrbRx_{Ch1}$$, $$OrbRx_{Ch2}$$, $$OrbRx_{Ch3}$$, generating the signals $$EncRx_{Ch1}$$, $$EncRx_{Ch2}$$, and $$EncRx_{Ch3}$$. The most significant difference between these transmitter and receiver is that the former includes the CSK modulation of the signals, so that when the signals of the receiver are subtracted from those of the transmitter, the bits that correspond to the encrypted information or the original data transmitted are exposed. The information decryption process consists of an X-OR between the signals $$EncRx_{Ch1}$$, $$EncRx_{Ch2}$$, $$EncRx_{Ch3}$$ and the pseudo-random signals generated in the receiver $$(SnlRx_x$$, $$SnlRx_y$$, $$SnlRx_z)$$. The result will be the original information $$(Data_{Ch1}$$, $$Data_{Ch2}$$, $$Data_{Ch3})$$.

**Figure 6:**
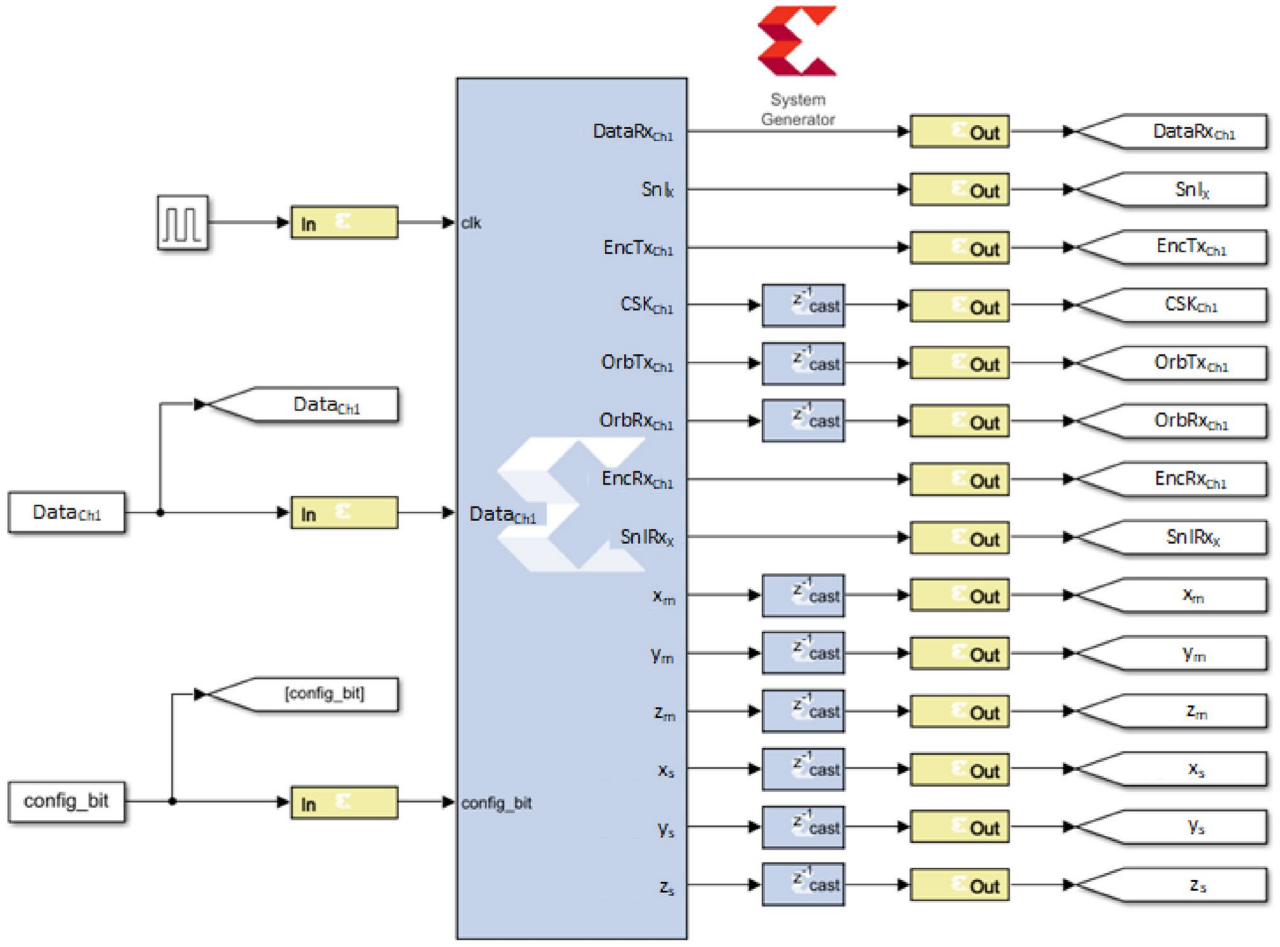
Co-simulation block diagram of the system proposed in this research work for the encryption of images in RGB and grayscale format, in Matlab-Simulink and Xilinx’s System Generator.

Co-simulation using Xilinx’s Matlab/Simulink System Generator for DSP plugin allows testing a design in VHDL language by interacting with pre-processed images, and also sends the digitized information to the circuit inputs. Figure 6 shows the complete system in a co-simulation between Vivado and MATLAB/Simulink represented by block diagrams. This diagram contains the essential blocks and signals for the process of encryption and modulation of RGB and grayscale images in the Xilinx Artix-7 AC701 card. It is worth mentioning that due to the portability of the source code, this same system can be used for the transmission and encryption of images for the Intel Cyclone IV board.

## Application in image transmission

**Figure 7:**
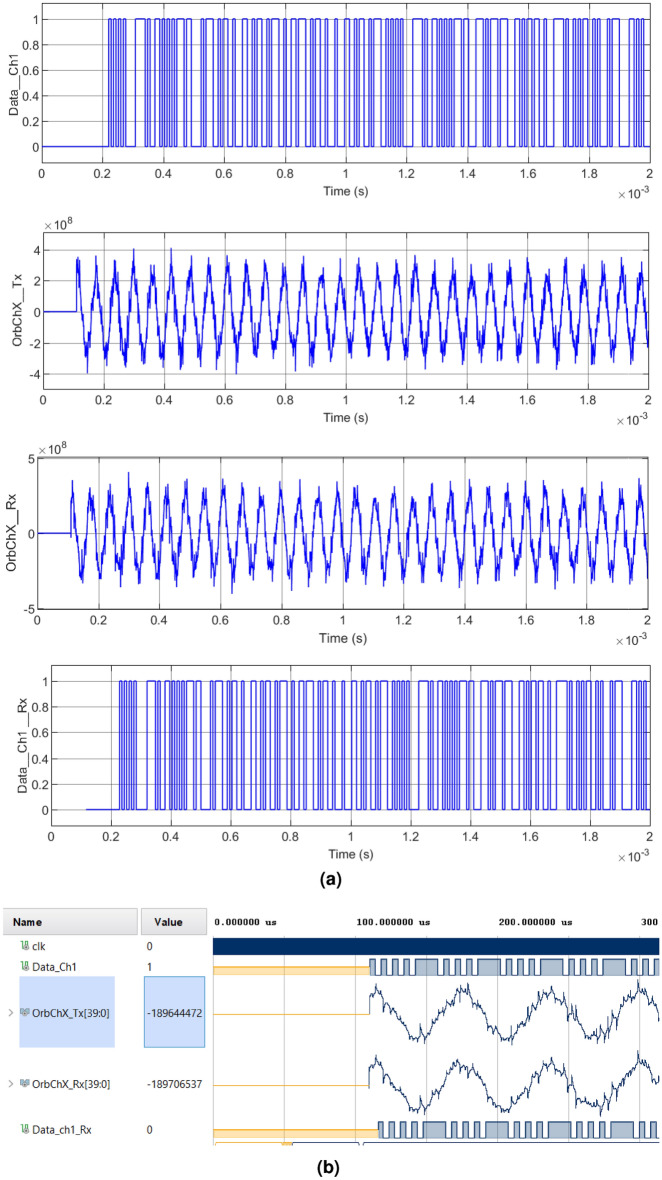
Modulation and demodulation process of the transmitted signal with chaotic interference of Case 1: (**a**) Matlab-Simulink. Upper left: $Data\_Ch1\_Tx$, Upper right: $OrbChX\_Tx$, Lower left: $OrbChX\_Rx$, Lower right: $Data \_Ch1\_Rx$, (**b**) Equivalent simulation in Vivado.

**Figure 8:**
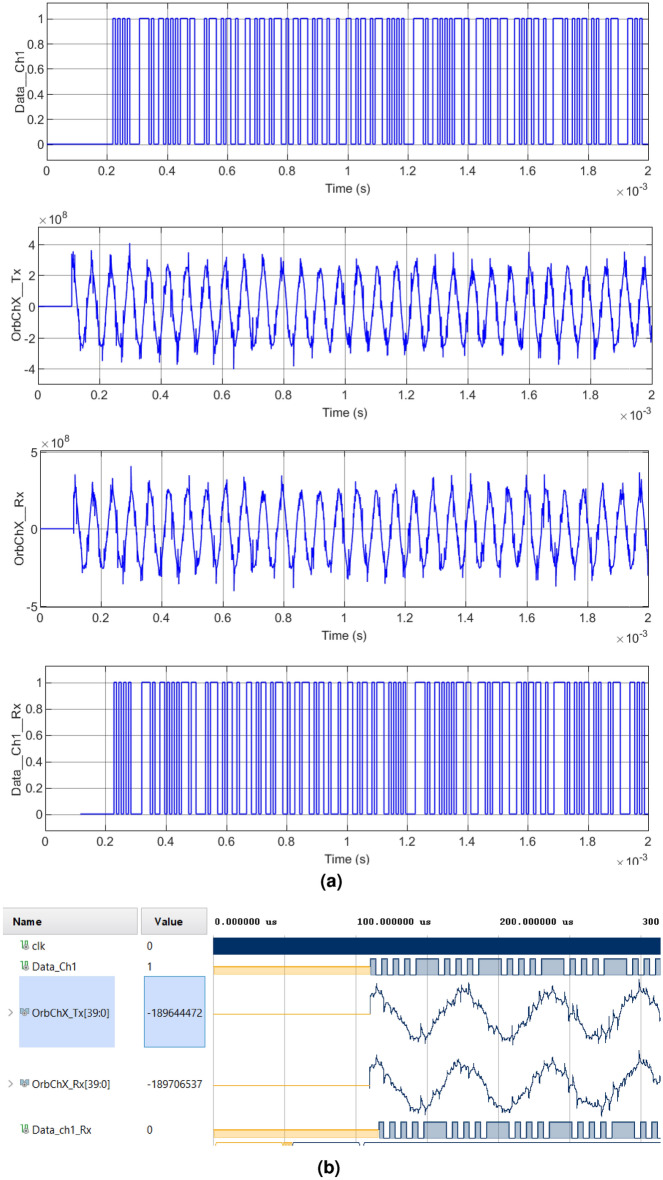
Modulation and demodulation process of the transmitted signal with chaotic interference of Case 2: (**a**) Matlab-Simulink. Upper left: $Data\_Ch1\_Tx$, Upper right: $OrbChX\_Tx$, Lower left: $OrbChX\_Rx$, Lower right: $Data \_Ch1\_Rx$, (**b**) Equivalent simulation in Vivado.

**Figure 9:**
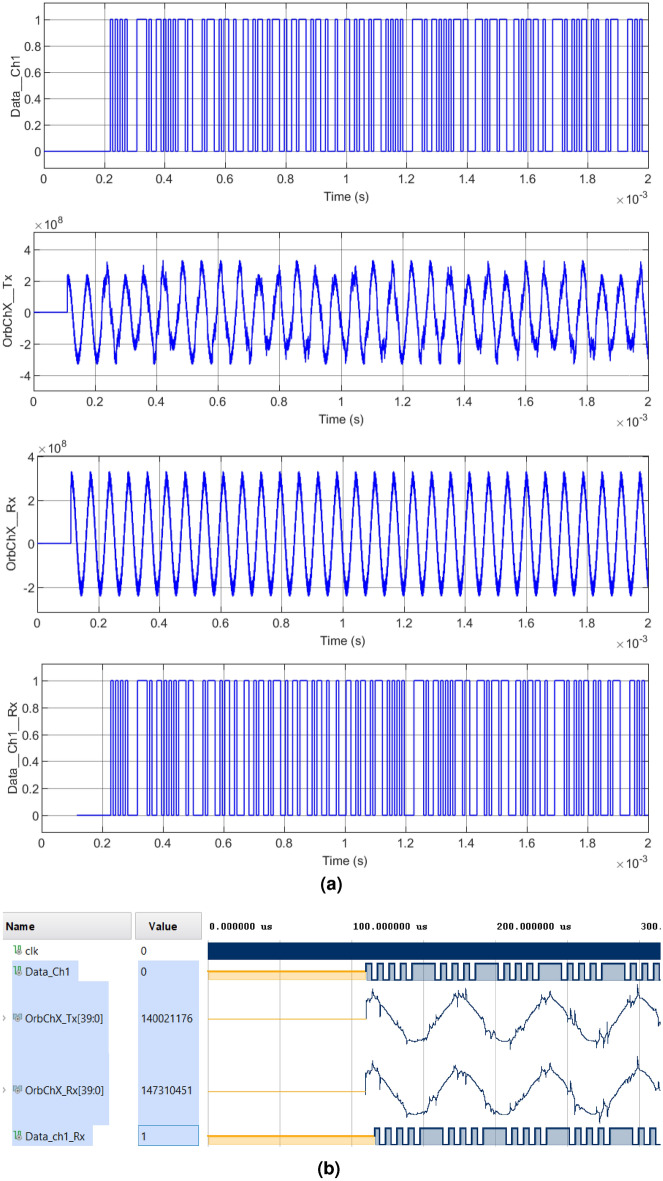
Modulation and demodulation process of the transmitted signal with chaotic interference in Case 3: (**a**) Matlab-Simulink. Upper left: $Data\_Ch1\_Tx$, Upper right: $OrbChX\_Tx$, Lower left: $OrbChX\_Rx$, Lower right: $Data \_Ch1\_Rx$, (**b**) Equivalent simulation in Vivado.

**Figure 10:**
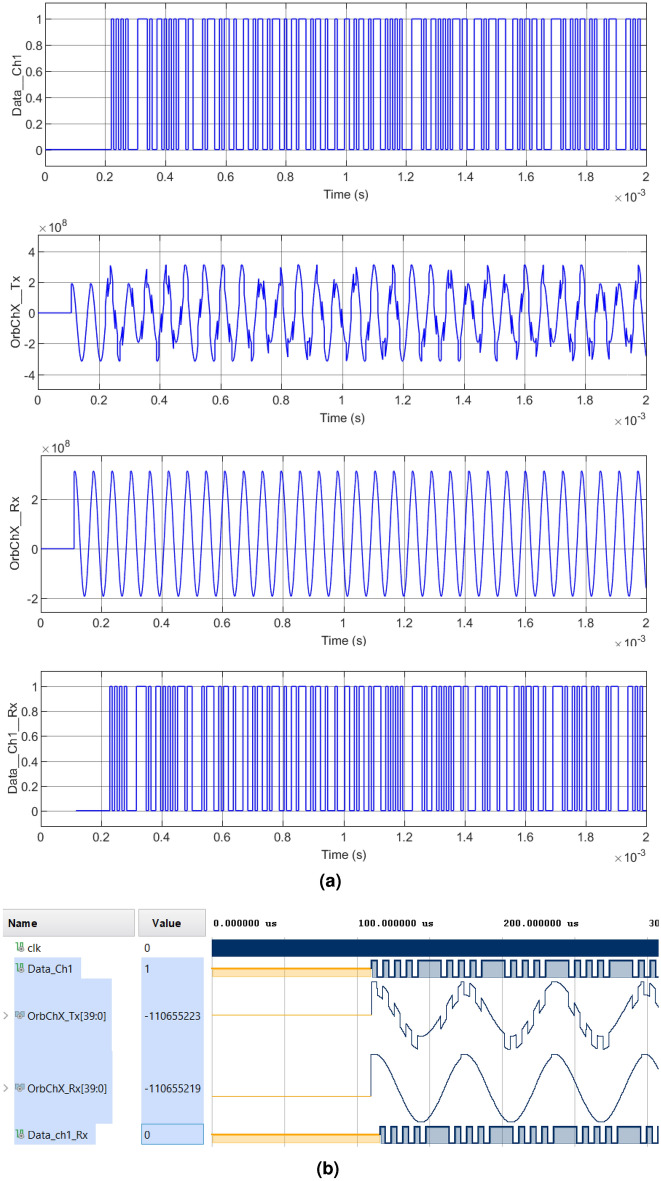
Modulation and demodulation process of the transmitted signal with chaotic interference from Case 4: (**a**) Matlab-Simulink. Upper left: $Data\_Ch1\_Tx$, Upper right: $OrbChX\_Tx$, Lower left: $OrbChX\_Rx$, Lower right: $Data \_Ch1\_Rx$, (**b**) Equivalent simulation in Vivado.

As a demonstration of the operation of the encryption and modulation system, described in Section “VHDL implementation and system co-simulation”, an RGB and a grayscale image were processed, with dimensions $320 \times 240\times 3$ pixels (230,400 samples) and $512 \times 512$ pixels (262,144 samples), respectively. The application in transmission-reception of images is briefly described in the following seven-step methodology: (1) Synchronizing the master and slave systems using the Hamiltonian technique and generating the RGB and grayscale image data files in VHDL; (2) Mixing the pseudo-random signals with the image data using an X-OR gate; (3) Adapting the chaotic data of the master system to the selected chaotic interference case to generate the carrier of the CSK modulator; (4) Applying the CSK modulation using the encrypted image signal as modulator and the chaotic signal of the interference case as carrier; (5) Mixing the output of the CSK modulator with the Earth’s orbital path data; (6) From the synchronization between master and slave, apply CSK demodulation of the received signal, and subtract the chaotic components and the case of interference; (7) Retrieving the image data, i.e., $DataRx_{Ch1}$, from the encrypted signal by removing chaos using the corresponding pseudo-random state of the slave system, i.e., $snlRx_x$;

In Figs. 7, 8, 9 and 10 we can see the verification of the functionality of the system proposed in this research work for cases $1-4$, respectively. The four cases illustrate the same digital and analog signals for contrast. Figure 7 shows Case 1, where the original information from an image channel $Data_{Ch1}$ is used as a modulator, $OrbTx_{ChX}$ corresponds to the modulated signal composed by the addition of the sum of the orbit, plus the chaotic interference and finally applying the CSK modulation. On the other hand, the receiver generates a signal composed by the addition of the chaotic signal with the Earth’s orbit but in this case without the CSK modulation called $OrbRx_{ChX}$, and serves as a reference for obtaining information from the subtraction of the signal $OrbTx_{ChX}$ to $OrbRx_{ChX}$. Finally $DataRx_{Ch1}$ corresponds to the information recovered from the demodulation of the received signal $OrbTx_{ChX}$. In this simulation the first 110 $\mu$ $s$ are used for the modulator to configure the oscillator parameters, then the encryption and modulation processes begin. Firstly, Fig. 7a illustrates the results obtained with the encryption of a segment of the message signal, a color channel of the modulated and demodulated RGB image using Matlab-Simulink. On the other hand, Fig. 7b shows the message signal, modulated and demodulated in the Xilinx Vivado software.

In Case 2, illustrated in Fig. 8, the periods vary their interference amplitude by 100% and 12.5%. In Case 3, shown in Fig. 9, the waveforms do not present a chaotic behavior, characteristic of the four-wing oscillator, instead, periodic oscillations are shown that develop along the orbit. In Case 4, shown in Fig. 10, it is clearly seen how the waveforms have been completely damped, due to the change in amplitude in the parameter *c*. In addition, it was possible to see that the results obtained in the encryption and transmission of images in Matlab and VHDL in Figs. 7, 8, 9 and 10 are the same. The time scale in the VHDL results in Vivado is expressed in microseconds ($\mu s$). The total simulation time was only 2 ms. However, it is possible to use the minimum clock period allowed by the FPGA, 5 ns for both Artix-7 and Cyclone IV boards, and thus further optimizing data processing times.

**Figure 11:**
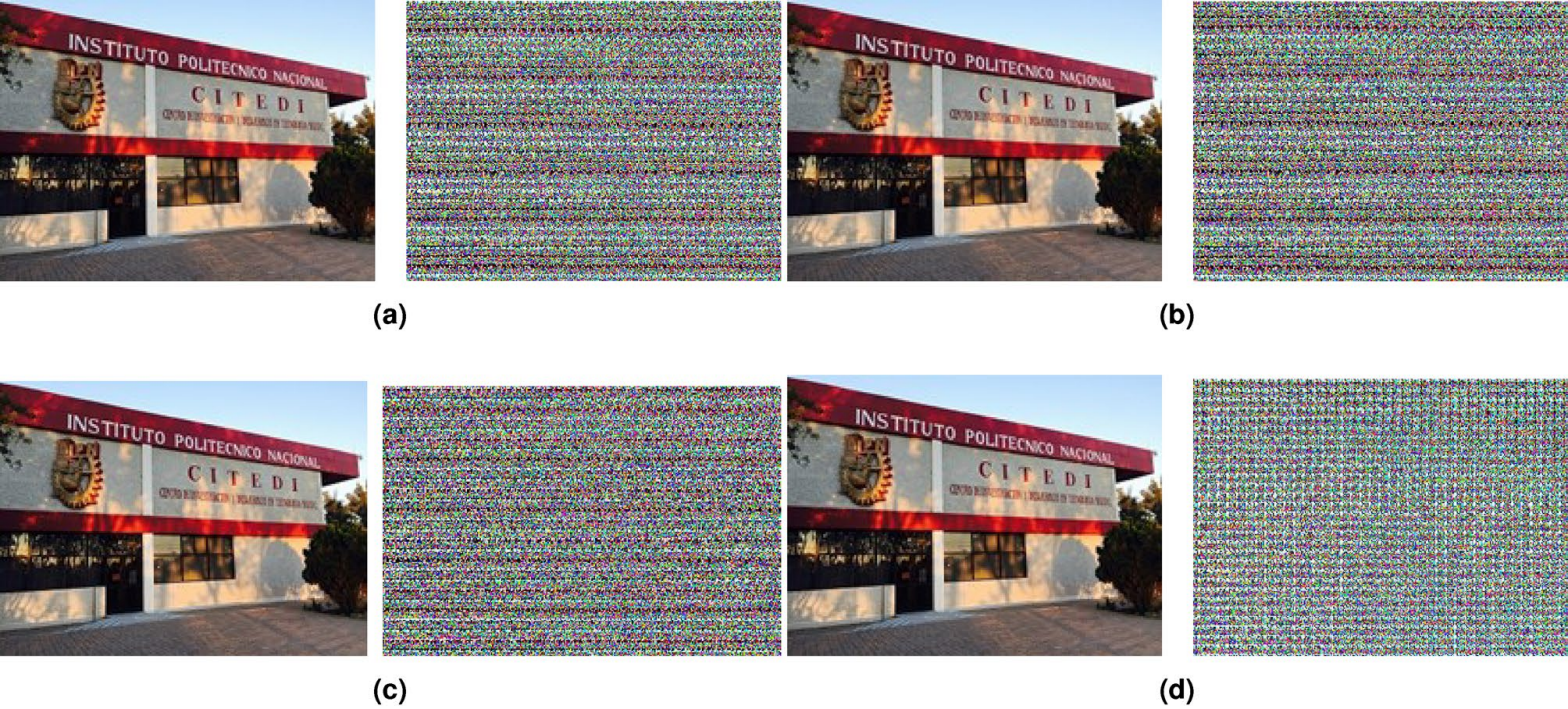
RGB image, original vs encrypted (**a**) Case 1, (**b**) Case 2, (**c**) Case 3, (**d**) Case 4.

**Figure 12:**
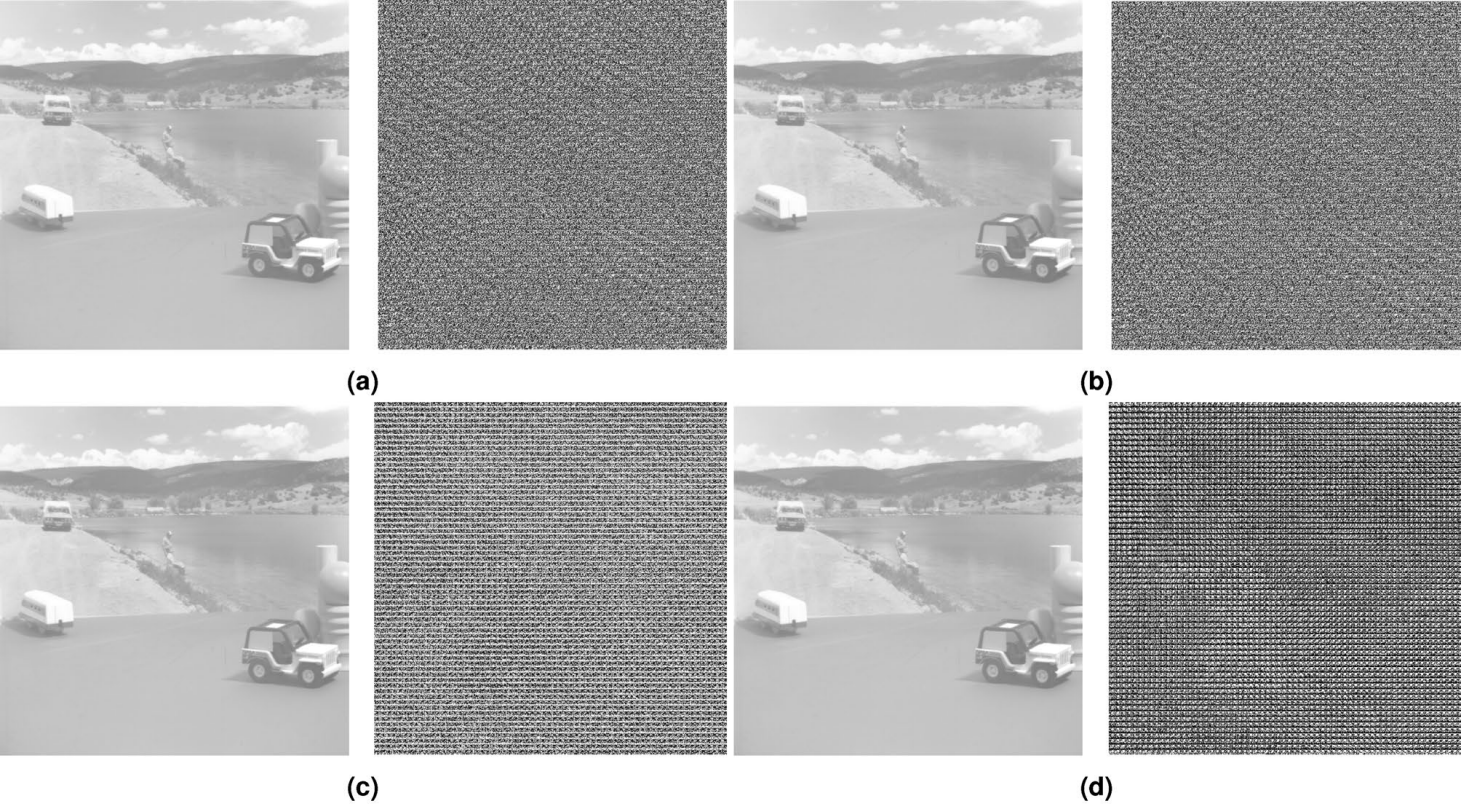
Grayscale image, original vs. encrypted (**a**) Case 1, (**b**) Case 2, (**c**) Case 3, (**d**) Case 4. [Grayscale image original: motion10.512, Toy Vehicle, frame 10, https://sipi.usc.edu/database/database.php?volume=sequences, 02/27/2023].

Figures 11 and 12 show the best encryption results for RGB and grayscale images, respectively, contrasting the four cases used in this research work.

The results of the chaotic image transmission system were examined with statistical analysis metrics. The statistical tests applied are information entropy and correlation. Table 1 shows the correlation and entropy coefficients obtained by the encrypted images with each of the four cases presented.

**Table 1:** Correlation coefficients and entropy between the original and encrypted RGB and grayscale image.

Cases	Transmission variable	Correlation	RGB image			Grayscale image
			Red	Green	Blue	
1	*x*	Original–encrypted	− 0.0044	− 0.00014	− 0.00017	− 0.0003439
		Original–received	1	1	1	1
		Encrypted entropy	7.8788	7.7889	7.6943	7.7198
	*y*	Original–encrypted	0.0014	0.00073	0.0010	0.0054
		Original–received	1	1	1	1
		Encrypted entropy	7.8799	7.7923	7.6970	7.6517
	*z*	Original–encrypted	− 0.0036	− 0.0103	− 0.0081	− 0.0019
		Original–received	1	1	1	1
		Encrypted entropy	7.8787	7.7920	7.6960	7.690
2	*x*	Original–encrypted	− 0.0044	− 0.000142	− 0.000173	− 0.0003439
		Original–received	1	1	1	1
		Encrypted entropy	7.8788	7.7889	7.6943	7.7198
	*y*	Original–encrypted	− 0.0014	0.000731	0.0010	0.0054
		Original–received	1	1	1	1
		Encrypted entropy	7.8799	7.7923	7.6970	7.6517
	*z*	Original–encrypted	− 0.0036	0.0103	− 0.0081	− 0.0019
		Original–received	1	1	1	1
		Encrypted entropy	7.8787	7.7920	7.6960	7.69
3	*x*	Original–encrypted	0.0108	− 0.0117	-0.0056	− 0.0022
		Original–received	1	1	1	1
		Encrypted entropy	7.8795	7.7923	7.6967	7.5498
	*y*	Original–encrypted	0.0054	0.0038	0.0025	− 0.005
		Original–received	1	1	1	1
		Encrypted entropy	7.8759	7.7813	7.6851	7.6065
	*z*	Original–encrypted	0.0134	− 0.0074	0.0048	− 0.0091
		Original–received	1	1	1	1
		Encrypted entropy	7.8700	7.7746	7.6763	7.4464
4	*x*	Original–encrypted	− 0.0235	− 0.0064	− 0.0161	0.0075
		Original–received	1	1	1	1
		Encrypted entropy	7.8497	7.7371	7.6292	6.9841
	*y*	Original–encrypted	− 0.0235	− 0.0064	− 0.0161	0.0075
		Original–received	1	1	1	1
		Encrypted entropy	7.8497	7.7371	7.6292	6.9841
	*z*	Original–encrypted	− 0.0235	− 0.0064	− 0.0161	0.0075
		Original–received	1	1	1	1
		Encrypted entropy	7.8497	7.7371	7.6292	6.9841

In the image encryption application, the correlation coefficients corroborate the effectiveness of the method proposed in this work. Case 1 using the variable *x* for an RGB image yielded the lowest correlation coefficient − 0.000142, for encryption and with an entropy in the encrypted image of 7.7889, with 8 being an ideal value in terms of a random distribution of pixels. In grayscale format, a correlation of -0.0003439 was achieved, with an entropy of 7.7198.

## Results and discussion

The implementation of the four-wing oscillator, encryption, modulation, synchronization, and image transmission, was carried out in two FPGA devices. As stated above, the HDL used was VHDL and the digital block size was forty bits: the entire segment used nineteen bits; the fraction occupied twenty bits and one bit for the sign. FPGA emulations were performed on the Xilinx Artix-7 AC701 card device number XC7A200TFBG676-2 and Intel Cyclone IV card device number EP4CE115F29C8. The FPGA logic resources consumed by the four-winged chaotic oscillator implementation are presented in Table 2.

**Table 2:** Logic resources used by the four-wings chaotic oscillator on the Artix-7 AC701 (XC7A200TFBG676-2) and Cyclone IV (EP4CE115F29C8) boards.

FPGA resources	Artix-7 (XC7A200TFBG676)			Cyclone IV (EP4CE115F29C8)		
	Available	Used	Utilization (%)	Available	Used	Utilization (%)
LUTs	134,600	3523	2.62	114,480	4837	4.22
Registers	269,200	120	<1	114,480	240	<1
I/O Pins	400	121	30.25	529	121	22.87
RAM	13,140,000	0	0	3,981,312	0	0
DSPs	740	84	11.35	532	254	47.74

With respect to the four-wings chaotic oscillator, it can be noted that both in the Xilinx and Intel boards the total number of pins used are the same since the same number of bits is being used for the output signals. Furthermore, less than one percent of the total registers on the FPGA boards were required for implementation. Finally, the four-wings chaotic oscillator required less than 5000 LUTs on both boards.

In the same way, Table 3 presents the logical resources required for the realization of the proposed complete system involving the four cases of chaotic interference in the two FPGA cards. The Artix-7 board uses less than the maximum available and the same as the Intel Cyclone IV card. It can be noted that Case II is the one that requires a greater number of logical resources because it requires additional processes to generate the sub-attractors of the system periodically. Additionally, cases 3 and 4 are virtually the same, this is because the equations are the same, and only the amplitude of the parameter *c* is varied for the generation of both cases. Hence, the logical resource requirements are similar for these two cases.

**Table 3:** Logical resources for the four cases in the transmit, receive and synchronization modules on the Artix-7 AC701 and Cyclone IV boards.

FPGA	Artix-7 (XC7A200TFBG676)				Cyclone IV (EP4CE115F29C8)			
	LUT	Registers	DSP	I/O	LUT	Registers	DSP	I/O
Transmitter	3629	120	84	208	5118	240	254	219
Receiver	4623	120	104	207	6444	200	298	219
Synchronizer	7735	240	188	241	11,070	440	532	241

Table 4 shows the logical resources required to encrypt RGB and grayscale images on both the Artix-7 and Cyclone IV boards. On the Cyclone IV card 100% of the DSPs are used. In the other metrics, both cards do not exceed the resource margin for the four case studies. The results obtained in terms of logical resources show that it is feasible to implement this type of system in digital development boards.

**Table 4:** Logical resources for cases 1, 2, 3 and 4 with encryption for RGB and grayscale images on Artix-7 AC701 and Cyclone IV development boards.

Cases	Resource	Artix-7		Cyclone IV	
		RGB	Grayscale	RGB	Grayscale
Case 1	LUT	11,202 (8.32%)	10,819 (8.04%)	18,345 (16.02%)	15,404 (13.45%)
	Registers	240 (1%)	240 (1%)	240 (1%)	240 (1%)
	I/O	18 (4.5%)	19 (4.75%)	18 (3.40%)	19 (3.59%)
	RAM	0	0	0	0
	DSP	197 (26.62%)	210 (28.38%)	532 (100%)	532 (100%)
Case 2	LUT	11,416 (8.48%)	10,886 (8.09%)	21,963 (19.18%)	16,743(14.62%)
	Registers	240 (<1%)	240 (< 1%)	240	240
	I/O	18 (4.5%)	19 (4.75%)	18 (3.40%)	19 (3.59%)
	RAM	0	0	0	0
	DSP	227 (30.68%)	204 (27.57%)	532 (100%)	532 (100%)
Case 3	LUT	11,431 (8.49%)	10,816 (8.04%)	18,648 (16.28%)	15,748 (10.89%)
	Registers	240 (< 1%)	240 (< 1%)	240	240
	I/O	18 (4.5%)	99 (24.75%)	18 (3.40%)	19 (3.59%)
	RAM	0	0	0	0
	DSP	213 (28.78%)	210 (28.38%)	532 (100%)	532 (100%)
Case 4	LUT	11,431 (8.49%)	10,816 (8.04%)	18,640 (16.28%)	15,740 (13.74%)
	Registers	240 (< 1%)	240 (< 1%)	240	240
	I/O	18 (4.5%)	99 (24.75%)	18 (3.40%)	19 (3.59%)
	RAM	0	0	0	0
	DSP	213 (28.78%)	210 (28.38%)	532 (100%)	532 (100%)

The results of the proposed system were compared with other systems reported in literature, where different cards and bit extensions have been used for the calculations. Table 5 shows the comparison of the results of various FPGA realizations^14,18–22^ that report their logical resources in comparison with the two developed in this investigation. The required resources exceed the values obtained by the reported investigations, but in our work we used 40 bits to carry out the calculations and a more complex system is employed by mixing natural dynamics with a chaotic oscillator.

**Table 5:** Comparison of this work with other FPGA implementations for secure communications reported in the literature.

Ref	This work		^14^	^18^	^19^	^20^	^21^	^22^
FPGA	Artix 7	Cyclone IV/E	Zinq	Cyclone IV/GX	Virtex 2	Virtex 6	Virtex 7	Cyclone IV
LUT	8.32	4.22	43	2	<1	4	1	5
FF	<1	<1	25	<1	<1	2	<1	3
I/O	4.5	22.87	24	-	27.06	41	21	-
DSP	26.62	47.74	-	19.44	-	22	5	6
Algorithm	RK4	RK4	RK4	RK4	RK4	RK4	RK4	Euler
HDL	VHDL	VHDL	Verilog	VHDL	VHDL	VHDL	VHDL	Verilog
Bits	40	40	32	27	32	32	32	32

Table 6 compares the results of correlation between encrypted and original images of this investigation with respect to other related investigations. According to reported research^10–13^, the modeling of natural dynamic systems in conjunction with practical encryption systems achieve good correlation results with techniques that do not use natural systems^23–27^. However, in none of these references is the implementation of the designs in VHDL or in FPGA cards, as in this research work. It is worth mentioning that the four-wing spherical chaotic oscillator and the parameters used in this work were implemented from^5^. Therefore, the encryption capabilities can be improved if an optimization using evolutionary algorithms is considered, reducing the correlation coefficients and increasing the entropy of the encrypted information.

**Table 6:** Comparison with the state of the art the correlation coefficients of the original RGB and grayscale image transmission using a CSK modulation scheme.

References	RGB image			Grayscale image
	Red	Green	Blue	
This work	0.0014	− 0.000142	− 0.000142	− 0.0003439
^10^	N/A	N/A	N/A	0.00152
^11^	0.0041	0.0030	0.0031	N/A
^12^	0.0003	− 0.0016	0.0005	N/A
^13^	N/A	N/A	N/A	− 0.0050
^23^	0.0008	0.0039	0.0009	N/A
^24^	0.00296	0.00296	0.00296	N/A
^25^	− 0.00005	− 0.00005	− 0.00005	N/A
^26^	− 0.0008	− 0.0008	− 0.0008	N/A
^27^	0.00343	0.00343	0.00343	N/A

## Conclusions

In this research work, a procedure was presented that allowed mixing a chaotic oscillator with waveforms from another system that for practical purposes is not chaotic. From this development, four study cases were generated with significant differences between them that enabled them to be used as carriers in a modulation process for cases 1 and 2, which are chaotic, and for cases 3 and 4, which are periodic. The amplitude of the interference contained in the generated sinusoidal as well as the sampling periods of the chaotic signal, among other parameters, can be manipulated in order to improve signal encryption and conditioning.

The elliptical trajectories of cases 1 and 2 acquire a disturbance along their path, whose unpredictable behavior is very useful in the area of data encryption since the orbit can be configured so that the final amplitude oscillates in a certain range. The multiple orbits can be used to encrypt different data packets without mixing them. In cases 3 and 4, a periodic perturbation is provided in the orbits and the same chaotic oscillator is used, only changing the amplitude in the parameter *c*. The correlation coefficients show that despite having a periodic disturbance in the orbit it is possible to achieve low levels of encryption. This is largely due to the signal used to encrypt the original information from a technique based on a digital gate X-OR. Additionally, CSK modulation was used as a carrier in the orbit of the Earth’s path with the addition of chaotic interference that allows increasing the encryption capacity of the system. The simulation results of the information modulation and demodulation in Vivado Design Suite and Matlab/Simulink showed complete agreement. Finally, the application in the encryption of images presents the best combination of results against those reported in the literature in terms of correlation coefficient and logical resources in its realization in FPGA boards.

## Data Availability

The datasets generated and analyzed during the current study are not publicly available due to the confidentiality but are available from the corresponding author on reasonable request.
