# Author Correction: FPGA realization of four chaotic interference cases in a terrestrial trajectory model and application in image transmission

**DOI:** 10.1038/s41598-024-65284-x

**Published:** 2024-06-26

**Authors:** Miguel‑Angel Estudillo‑Valdez, Vincent‑Ademola Adeyemi, Esteban Tlelo‑Cuautle, Yuma Sandoval‑Ibarra, Jose‑Cruz Nuñez‑Perez

**Affiliations:** 1 Instituto Politécnico Nacional, CITEDI, 22435 Tijuana, Mexico; 2 Instituto Nacional de Astrofísica, Óptica y Electrónica, INAOE, 72840 San Andrés Cholula, Mexico; 3 Universidad Politécnica de Lázaro Cárdenas, UPLC, 60950 Lázaro Cárdenas, Mexico

Correction to: *Scientific Reports* 10.1038/s41598-023-39823-x, published online 10 August 2023

The original version of this Article contained an error in Reference 7, which was incorrectly given as:

Liu, Z., So, H. C., Zhang, L. & Li, X. P. Performance evaluation of IEEE 802.15.7 CSK modulation under blue shift effect of optical receiver filters. Phys. Commun. 49, 1–9. 10.1016/j.phycom.2021.101481 (2021).

The correct reference is listed below:

Küçük, M. A. & Türk, K. Performance evaluation of IEEE 802.15.7 CSK modulation under blue shift effect of optical receiver filters. Phys. Commun. 49, 1–9. 10.1016/j.phycom.2021.101481 (2021).

The original Article has been corrected.

